# Evaluating the clinical efficacy of a long-read sequencing-based approach for carrier screening of spinal muscular atrophy

**DOI:** 10.1186/s40246-024-00676-8

**Published:** 2024-09-29

**Authors:** Ju Long, Di Cui, Chunhui Yu, Wanli Meng

**Affiliations:** 1 Laboratory of Medical Genetics, Qinzhou Maternal and Child Health Care Hospital, Qinzhou, Guangxi Province 535099 China; 2 Berry Genomics Corporation, Beijing, 102200 China

**Keywords:** Spinal muscular atrophy (SMA), Multiplex ligation-dependent probe amplification (MLPA), Quantitative polymerase chain reaction (qPCR), Comprehensive SMA analysis (CASMA), Third-generation sequencing (TGS)

## Abstract

Spinal muscular atrophy (SMA) is the second most common fatal genetic disease in infancy. It is caused by deletion or intragenic pathogenic variants of the causative gene *SMN1*, which degenerates anterior horn motor neurons and leads to progressive myasthenia and muscle atrophy. Early treatment improves motor function and prognosis in patients with SMA, but drugs are expensive and do not cure the disease. Therefore, carrier screening seems to be the most effective way to prevent SMA birth defects. In this study, we genetically analyzed 1400 samples using multiplex ligation-dependent probe amplification (MLPA) and quantitative polymerase chain reaction (qPCR), and compared the consistency of the results. We randomly selected 44 samples with consistent MLPA and qPCR results for comprehensive SMA analysis (CASMA) using a long-read sequencing (LRS)-based approach. CASMA results showed 100% consistency, visually and intuitively explained the inconsistency between exons 7 and 8 copy numbers detected by MLPA in 13 samples. A total of 16 samples showed inconsistent MLPA and qPCR results for *SMN1* exon 7. CASMA was performed on all samples and the results were consistent with those of resampling for MLPA and qPCR detection. CASMA also detected an additional intragenic variant c.-39A>G in a sample with two copies of *SMN1* (RT02). Finally, we detected 23 SMA carriers, with an estimated carrier rate of 1/61 in this cohort. In addition, CASMA identified the “2 + 0” carrier status of *SMN1* and *SMN2* in a family by analyzing the genotypes of only three samples (parents and one sibling). CASMA has great advantages over MLPA and qPCR assays, and could become a powerful technical support for large-scale screening of SMA.

## Background

Spinal muscular atrophy (SMA) is a disability-causing autosomal recessive neuromuscular disease, characterized by muscle weakness and atrophy due to degeneration and loss of motor neurons in the anterior horn of the spinal cord [1]. Pathogenic variants in the survival motor neuron (*SMN*) gene cause defects in SMN protein function. Degeneration of α-motor neurons in the spinal cord leads to muscle weakness and muscle atrophy in the proximal skeleton, which causes progressive, symmetrical flaccid paralysis and muscular atrophy of the proximal limbs, respiratory disturbances, and movement disorders in patients [2].

**Fig. 1:**
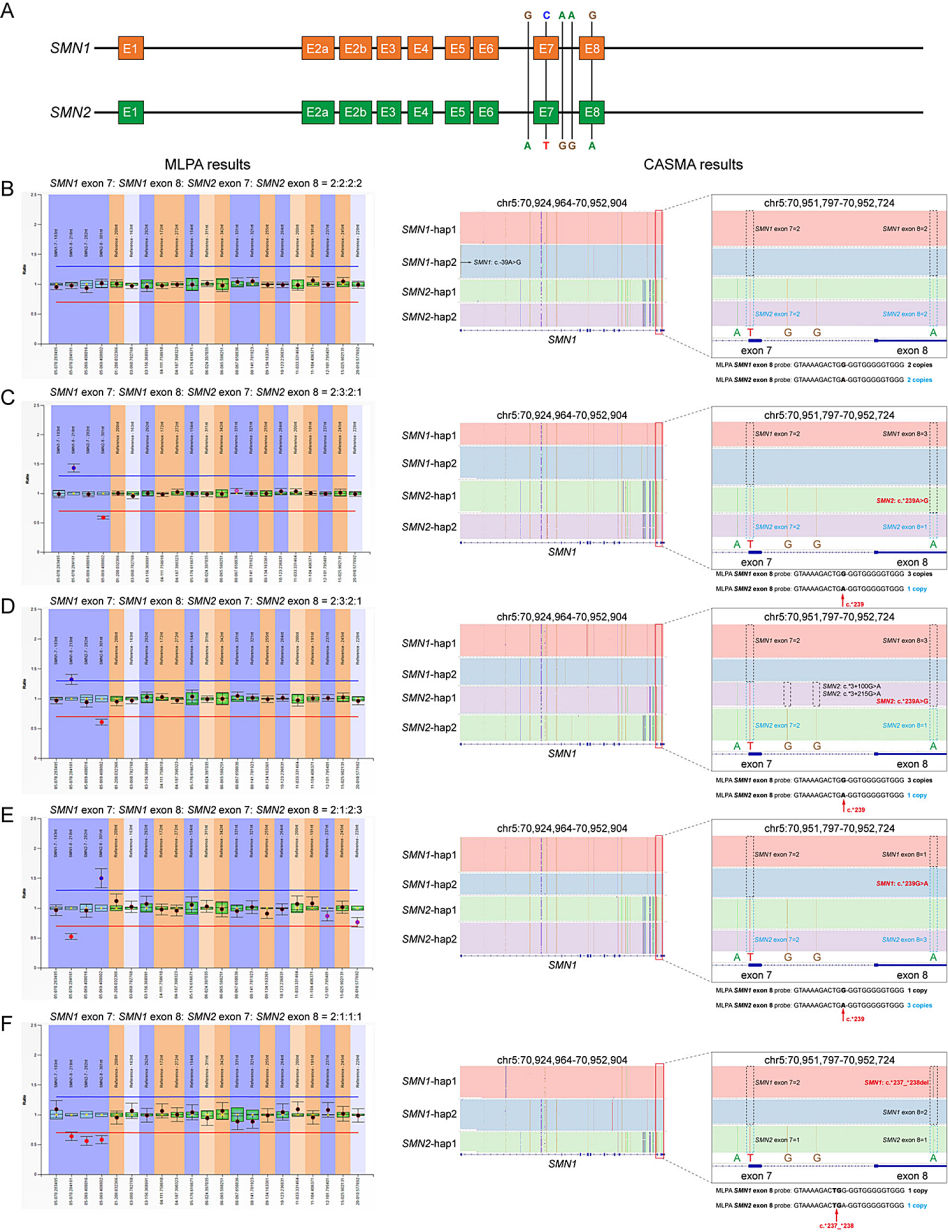
Samples with inconsistent copy numbers of *SMN1* exons 7 and 8 were determined by MLPA and CASMA. (**A**) Presentation of full-length *SMN* genes. Five paralogous sequence variants (PSVs) between *SMN1* and *SMN2* were labeled on the diagram. (**B**-**F**) The left and right panels showed the results of MLPA and CASMA, respectively. Grey boxes showed the enlarged CASMA results. Sequences of MLPA *SMN* exon 8 probes, copy numbers of *SMN* exon 8, and locations of c.*239, c.*237_238 were shown below grey boxes. (**B**) A normal MLPA result with *SMN1* exon 7: *SMN1* exon 8: *SMN2* exon 7: *SMN2* exon 8 = 2:2:2:2. A variant c.-39A>G was identified in one copy of *SMN1* (RT02). (**C**) YZ38 had one *SMN2* variant c.*239A>G. The MLPA result was *SMN1* exon 7: *SMN1* exon 8: *SMN2* exon 7: *SMN2* exon 8 = 2:3:2:1. (**D**) YZ32 had *SMN2* variants c.*3+100A> C, c.*3+215A>G, and c.*239A>G. The MLPA result was *SMN1* exon 7: *SMN1* exon 8: *SMN2* exon 7: *SMN2* exon 8 = 2:3:2:1. (**E**) YZ42 had *SMN1* variant c.*239G>A. The MLPA result was *SMN1* exon 7: *SMN1* exon 8: *SMN2* exon 7: *SMN2* exon 8 = 2:1:2:3. (**F**) YZ44 had one *SMN1* variant c.*237_*238del. The MLPA result was *SMN1* exon 7: *SMN1* exon 8: *SMN2* exon 7: *SMN2* exon 8 = 2:1:1:1, of which the actual copy number of *SMN1* exon 8 was 2

The *SMN* gene is located in the q13 region of chromosome 5 and contains two highly homologous genes, *SMN1* and *SMN2*. The two genes are > 90% homologous, with only a five-base difference near exons 7 and 8, of which the one-base difference c.840C/T on exon 7 mainly affects the function (Fig. 1A) [3]. The *SMN1* gene plays the primary function and determines the onset of disease. When the *SMN1* gene is ineffective, the *SMN2* gene acts as a modifier gene and influences the severity and progression of SMA disease [4]. The more copies of the normal *SMN2* gene a patient carries, the milder SMA phenotype the patient will exhibit. Therefore, when making a genetic diagnosis, clinicians would test the copy number of exon 7 of both *SMN1* and *SMN2* to determine whether they are SMA patients and to initially determine the possible severity of their disease.

The incidence of SMA is about 1/10,000, and the carrier rate in the population is about 1/50 [5]. SMA is usually categorized into four clinical types based on age of onset and highest motor function achieved, with an additional phenotype (type 0) describing the severe form of prenatal-onset SMA [6]. Types 0–2 diseases are severe and common types, which accounts for more than 80% of all SMA cases [7]. If left untreated, patients tend to die in the first few days of life (type 0), before the age of 2 years (type 1) and in adulthood (type 2) [7]. Early treatment improves motor function and prognosis in patients with SMA, but drugs are expensive and do not cure the disease [8]. Carrier screening and prenatal diagnosis for couples of childbearing age to prevent SMA birth defects appears to be a more effective mean of preventing and controlling SMA. About 95% of SMA patients are caused by homozygous deletion of *SMN1* exon 7 [9, 10]. And the genotype of about 95% of SMA carriers is the heterozygous deletion of *SMN1* exon 7 [11]. Therefore, genetic testing for SMA carriers mainly focuses on the detection of the copy number of *SMN1* exon 7. At present, multiplex ligation probe amplification (MLPA) and fluorescence quantitative PCR (qPCR) are the most commonly used methods to determine the copy number of *SMN1* [12, 13]. The MLPA method designs hybridization probes for the base difference sites of exons 7 and 8 of *SMN1* and *SMN2* genes (C/T at locus c.840 and G/A at locus c.*239) and employs multiple housekeeping genes from other chromosome loci as internal reference genes. Samples with different copy numbers of *SMN1* and *SMN2* are used as parallel controls. After hybridization and linking, the copy number of the target gene sequence is determined according to the ratio of the fluorescence peak area. MLPA can differentiate between patients, carriers, and normal people by directly detecting the copy number of *SMN1*, and can also detect the copy number of *SMN2* in subjects at the same time. MLPA is the gold standard for diagnosing SMA, which is recommended by the SMA management consensus in several countries [14]. The fluorescence quantitative PCR method (qPCR) is a combination of multiplex real-time fluorescence quantitative PCR and multicolor Taqman fluorescent probe technology. In this method, the housekeeping gene sequence is used as the internal reference, and the relative quantitation of the copy numbers of *SMN1* exons 7 and 8 is determined by comparing the Ct values. Both MLPA and qPCR are based on the principle of the probe method, which makes it difficult to accurately differentiate between *SMN1* and *SMN2* with only one nucleotide difference in exon 7. In particular, due to the high homology of *SMN1* and *SMN2*, homologous recombination during replication and segregation can lead to various complex structural variants [15]. A typical complex structural variant is that some individuals have two *SMN1* genes in *cis* arrangement on the same chromosome. Individuals carrying this type of variant are called silent “2 + 0” carriers and are at risk of having children with SMA when their spouse is also an SMA carrier.

In this study, MLPA and qPCR assays were used to determine the copy number of *SMN* genes in 1400 samples and to compare the consistency of the results. For the samples with consistent MLPA and qPCR results, the emerging long-read sequencing (LRS)-based approach called comprehensive SMA analysis (CASMA) was performed on 44 samples to evaluate its detection performance [16]. For the 16 samples with inconsistent results for *SMN1* exon 7, CASMA was also performed to analyze the possible reasons for the inconsistency. The CASMA results were comprehensively compared with the first two commonly used testing methods to explore and analyze the actual clinical efficacy in screening for SMA carriers.

## Methods

### Subjects

A total of 1400 subjects of childbearing age who came to our hospital for carrier screening during July 2023 to December 2023 (male to female ratio was about 1:1) and seven samples from one family were enrolled in this study. Informed consent was obtained from all participants or their legal guardians involved in the study.

### DNA extraction

DNA was extracted and measured using the Blood Genomic DNA Mini Kit (Cwbio, Beijing, China) and NanoDrop spectrophotometer, respectively. DNA was stored in an environment of -20℃ before use.

### MLPA analysis

The SALSA^®^ MLPA^®^ Probemix P060-B2 SMA Carrier (MRC-Holland, Amsterdam, The Netherlands) was used in this study. The kit was completed according to the manufacturer’s instructions. After denaturation, hybridization, probe connection, and PCR, a 3500xL Dx genetic analyzer (Applied Biosystems, Foster City, CA) was used for detection. The test data were imported into Coffalyser (v.220513, MRC Holland, Amsterdam, The Netherlands) for further analysis.

### qPCR analysis

The Motor Neuron Survival Gene 1 (*SMN1*) Exon Deletion Detection Kit (Wuse Shi Medical Technology, Shanghai, China) was used for the qPCR assay in this study. Exons 7 and 8 of *SMN1* were amplified and relatively quantified. And chemical blocking was used to control the effect of *SMN2* on the detection results. The test was performed on the Hongshi SLAN96S real-time fluorescence analysis system (Hongshi, Shanghai, China) following the procedure: 95℃ for 10 min, 40 cycles of 95℃ for 15 s and 58℃ for 60 s. Fluorescence was collected after each cycle. In this study, two samples with two-copy *SMN1* were added to each assay as a reference, and the relative copy number could be roughly analyzed using the 2^−ΔΔCq^ method.

### CASMA analysis

The full-length sequence of *SMN1*/*2* was determined by the method named CASMA based on the LRS (the third-generation sequencing, TGS) as previously described [17]. Briefly, the full-length sequence of the *SMN1* and *SMN2* genes was amplified (KOD FX Neo Polymerase, TOYOBO, Osaka, Japan) and ligated by a unique hairpin barcode adapter to form a dumbbell-shaped pre-library. Exonuclease (Enzymatics, Beverly, MA) was then added to remove failed DNA ligations. After purification and quantification, equal mass was pooled to form single-molecule real-time dumbbell (SMRTbell) libraries. SMRTbell libraries were prepared using the Sequel II Binding Kit 3.2 (Pacific Biosciences, CA, US) and then sequenced on the Sequel IIe platform (Pacific Biosciences, CA, US) for 30 h using the cyclic consensus sequencing (CCS) mode.

Raw subreads of each sample were debarcoded and aligned to the hg38 reference using lima (in the Pbbioconda package, smrtlink 10.1.0.119588, Pacific Biosciences) and pbmm2 (version 1.5.0), respectively. *SMN1* and *SMN2* genes were differentiated by c.840. For haplotype analysis, each CCS read was aligned with the internal reference gene to obtain all SNPs using FreeBayes version 1.3.4 (Biomatters, Inc.,San Diego, CA). Only CCS reads whose SNPs frequencies between 20% and 80% were retained for haplotype determination. These CCS reads were recursively divided into two groups by SNPs until no further division was possible [17]. Each final group was a specific haplotype, and the number of reads per haplotype was counted. The copy numbers of *SMN1* and *SMN2* were determined using a Poisson distribution-based caller with the haplotype numbers and read count as inputs [16]. CCS reads of representative samples were displayed in the Integrated Genomics Viewer (IGV) to show the different haplotypes of *SMN1* and *SMN2*.

### Short tandem repeat (STR) analysis

STR analysis was used to determine the relationships among family members. Multiple STRs were analyzed using the Goldeneye™ DNA ID System 20 A (Beijing PeopleSpot Inc, Beijing, China) with 19 target loci of *D19S433*, *D5S818*, *D21S11*, *D18S51*, *D6S1043*, *D3S1358*, *D13S317*, *D7S820*, *D16S539*, *CSF1PO*, *Penta D*, *vWA*, *D8S1179*, *TPOX*, *Penta E*, *TH01*, *D12S391*, *D2S1338*, and *FGA*. Sex was determined using characterized sequences in Amelogenin. DNA from each sample was amplified using the Applied Biosystems Veriti instrument (Applied Biosystems, Foster City, CA), and was sequenced on the 3500xL Dx genetic analyzer (Applied Biosystems, Foster City, CA). Finally, the assay data were imported into the GeneMapper^®^ ID-X (Applied Biosystems, Foster City, CA) to complete the analysis.

## Results

### Samples with consistent MLPA and qPCR results

MLPA and qPCR testing were used to analyze the copy numbers of *SMN1* and *SMN2* carried by each subject. In this study, samples with MLPA test results showing *SMN1* exon 7: *SMN1* exon 8: *SMN2* exon 7: *SMN2* exon 8 = 2:2:2:2 were defined as negative samples, samples with other test results were defined as positive samples. Forty-four samples were randomly selected from the 676 positive samples whose MLPA and qPCR results were consistent. CASMA was performed on these samples to evaluate the detection performance (Table 1). For exon 7 of *SMN1* and *SMN2*, the test results of CASMA were consistent with those of MLPA and qPCR. Among the selected samples, the copy numbers of *SMN1* exons 7 and 8 were inconsistent in 13 samples (YZ32- YZ44). According to the CASMA analysis, the conversion between *SMN1* and *SMN2* occurred in 12 samples, among which the conversion region of 11 samples was exon 8 (YZ33-YZ43, Fig. 1C and D), and the conversion region of one sample included part of intron 7 and exon 8 (YZ32, Fig. 1E). The other sample had a variant in the region where the MLPA probe binds (YZ44, Fig. 1F). The MLPA result was *SMN1* exon 7: *SMN1* exon 8: *SMN2* exon 7: *SMN2* exon 8 = 2:1:1:1, of which the actual copy number of *SMN1* exon 8 was 2.

**Table 1:** CASMA analysis of samples with consistent MLPA and qPCR results

Sample	MLPA SMN1		MLPA SMN2		qPCR SMN1		CASMA SMN1			CASMA SMN1			Comments
	Exon 7	Exon 8	Exon 7	Exon 8	Exon 7	Exon 8	Specific PSVs^a^	Exon 7	Exon 8	Specific PSVs^a^	Exon 7	Exon 8	
YZ01	1	1	1	1	1	1	G*C*AA*G*, *n* = 1	1	1	A*T*GG*A*, *n* = 1	1	1	Consistent^b^
YZ02	1	1	3	3	1	1	G*C*AA*G*, *n* = 1	1	1	A*T*GG*A*, *n* = 3	3	3	Consistent^b^
YZ03	1	1	3	3	1	1	G*C*AA*G*, *n* = 1	1	1	A*T*GG*A*, *n* = 3	3	3	Consistent^b^
YZ04	1	1	3	3	1	1	G*C*AA*G*, *n* = 1	1	1	A*T*GG*A*, *n* = 3	3	3	Consistent^b^
YZ05	1	1	3	3	1	1	G*C*AA*G*, *n* = 1	1	1	A*T*GG*A*, *n* = 3	3	3	Consistent^b^
YZ06	1	1	3	3	1	1	G*C*AA*G*, *n* = 1	1	1	A*T*GG*A*, *n* = 3	3	3	Consistent^b^
YZ07	2	2	1	1	2	2	G*C*AA*G*, *n* = 2	2	2	A*T*GG*A*, *n* = 1	1	1	Consistent^b^
YZ08	3	3	0	0	3	3	G*C*AA*G*, *n* = 3	3	3	A*T*GG*A*, *n* = 0	0	0	Consistent^b^
YZ09	3	3	1	1	3	3	G*C*AA*G*, *n* = 3	3	3	A*T*GG*A*, *n* = 1	1	1	Consistent^b^
YZ10	3	3	1	1	3	3	G*C*AA*G*, *n* = 3	3	3	A*T*GG*A*, *n* = 1	1	1	Consistent^b^
YZ11	3	3	1	1	3	3	G*C*AA*G*, *n* = 3	3	3	A*T*GG*A*, *n* = 1	1	1	Consistent^b^
YZ12	3	3	1	1	3	3	G*C*AA*G*, *n* = 3	3	3	A*T*GG*A*, *n* = 1	1	1	Consistent^b^
YZ13	3	3	1	1	3	3	G*C*AA*G*, *n* = 3	3	3	A*T*GG*A*, *n* = 1	1	1	Consistent^b^
YZ14	3	3	1	1	3	3	G*C*AA*G*, *n* = 3	3	3	A*T*GG*A*, *n* = 1	1	1	Consistent^b^
YZ15	3	3	1	1	3	3	G*C*AA*G*, *n* = 3	3	3	A*T*GG*A*, *n* = 1	1	1	Consistent^b^
YZ16	3	3	1	1	3	3	G*C*AA*G*, *n* = 3	3	3	A*T*GG*A*, *n* = 1	1	1	Consistent^b^
YZ17	3	3	1	1	3	3	G*C*AA*G*, *n* = 3	3	3	A*T*GG*A*, *n* = 1	1	1	Consistent^b^
YZ18	3	3	1	1	3	3	G*C*AA*G*, *n* = 3	3	3	A*T*GG*A*, *n* = 1	1	1	Consistent^b^
YZ19	3	3	1	1	3	3	G*C*AA*G*, *n* = 3	3	3	A*T*GG*A*, *n* = 1	1	1	Consistent^b^
YZ20	3	3	1	1	3	3	G*C*AA*G*, *n* = 3	3	3	A*T*GG*A*, *n* = 1	1	1	Consistent^b^
YZ21	3	3	1	1	3	3	G*C*AA*G*, *n* = 3	3	3	A*T*GG*A*, *n* = 1	1	1	Consistent^b^
YZ22	3	3	3	3	3	3	G*C*AA*G*, *n* = 3	3	3	A*T*GG*A*, *n* = 3	3	3	Consistent^b^
YZ23	3	3	3	3	3	3	G*C*AA*G*, *n* = 3	3	3	A*T*GG*A*, *n* = 3	3	3	Consistent^b^
YZ24	3	3	2	2	3	3	G*C*AA*G*, *n* = 3	3	3	A*T*GG*A*, *n* = 2	2	2	Consistent^b^
YZ25	3	3	2	2	3	3	G*C*AA*G*, *n* = 3	3	3	A*T*GG*A*, *n* = 2	2	2	Consistent^b^
YZ26	3	3	2	2	3	3	G*C*AA*G*, *n* = 3	3	3	A*T*GG*A*, *n* = 2	2	2	Consistent^b^
YZ27	3	3	2	2	3	3	G*C*AA*G*, *n* = 3	3	3	A*T*GG*A*, *n* = 2	2	2	Consistent^b^
YZ28	3	3	2	2	3	3	G*C*AA*G*, *n* = 3	3	3	A*T*GG*A*, *n* = 2	2	2	Consistent^b^
YZ29	3	3	2	2	3	3	G*C*AA*G*, *n* = 3	3	3	A*T*GG*A*, *n* = 2	2	2	Consistent^b^
YZ30	3	3	2	2	3	3	G*C*AA*G*, *n* = 3	3	3	A*T*GG*A*, *n* = 2	2	2	Consistent^b^
YZ31	4	4	1	1	4	4	G*C*AA*G*, *n* = 4	4	4	A*T*GG*A*, *n* = 1	1	1	Consistent^b^
YZ32	2	3	2	1	2	3	G*C*AA*G*, *n* = 2	2	2	A*T*GG*A*, *n* = 1 A*T***AA*G***, *n* = 1	2	1 belongs to *SMN2* 1 belongs to *SMN1*	*SMN2* variants: c.*3+100A>C, c.*3+215A>G, c.*239A>G; 1 copy of *SMN2* exon 8 converted to *SMN1* exon 8
YZ33	2	3	2	1	2	3	G*C*AA*G*, *n* = 2	2	2	A*T*GG*A*, *n* = 1 A*T*GG***G***, *n* = 1	2	1 belongs to *SMN2* 1 belongs to *SMN1*	*SMN2* variant: c.*239A>G; 1 copy of *SMN2* exon 8 converted to *SMN1* exon 8
YZ34	2	3	2	1	2	3	G*C*AA*G*, *n* = 2	2	2	A*T*GG*A*, *n* = 1 A*T*GG***G***, *n* = 1	2	1 belongs to *SMN2* 1 belongs to *SMN1*	*SMN2* variant: c.*239A>G; 1 copy of *SMN2* exon 8 converted to *SMN1* exon 8
YZ35	2	3	2	1	2	3	G*C*AA*G*, *n* = 2	2	2	A*T*GG*A*, *n* = 1 A*T*GG***G***, *n* = 1	2	1 belongs to *SMN2* 1 belongs to *SMN1*	*SMN2* variant: c.*239A>G; 1 copy of *SMN2* exon 8 converted to *SMN1* exon 8
YZ36	2	3	2	1	2	3	G*C*AA*G*, *n* = 2	2	2	A*T*GG*A*, *n* = 1 A*T*GG***G***, *n* = 1	2	1 belongs to *SMN2* 1 belongs to *SMN1*	*SMN2* variant: c.*239A>G; 1 copy of *SMN2* exon 8 converted to *SMN1* exon 8
YZ37	2	3	2	1	2	3	G*C*AA*G*, *n* = 2	2	2	A*T*GG*A*, *n* = 1 A*T*GG***G***, *n* = 1	2	1 belongs to *SMN2* 1 belongs to *SMN1*	*SMN2* variant: c.*239A>G; 1 copy of *SMN2* exon 8 converted to *SMN1* exon 8
YZ38	2	3	2	1	2	3	G*C*AA*G*, *n* = 2	2	2	A*T*GG*A*, *n* = 1 A*T*GG***G***, *n* = 1	2	1 belongs to *SMN2* 1 belongs to *SMN1*	*SMN2* variant: c.*239A>G; 1 copy of *SMN2* exon 8 converted to *SMN1* exon 8
YZ39	3	4	1	0	3	4	G*C*AA*G*, *n* = 3	3	3	A*T*GG***G***, *n* = 1	1	1 belongs to *SMN1*	*SMN2* variant: c.*239A > G; 1 copy of *SMN2* exon 8 converted to *SMN1* exon 8
YZ40	3	2	1	2	3	2	G*C*AA*G*, *n* = 2 G*C*AA***A***, *n* = 1	3	2 belong to *SMN1* 1 belongs to *SMN2*	A*T*GG*A*, *n* = 1	1	1	*SMN1* variant: c.*239G>A; 1 copy of *SMN1* exon 8 converted to *SMN2* exon 8
YZ41	2	1	2	3	2	1	*GC*AAG, *n* = 1 *GC*AA***A***, *n* = 1	2	1 belongs to *SMN1* 1 belongs to *SMN2*	A*T*GG*A*, *n* = 2	2	2	*SMN1* variant: c.*239G>A; 1 copy of *SMN1* exon 8 converted to *SMN2* exon 8
YZ42	2	1	2	3	2	1	G*C*AA*G*, *n* = 1 G*C*AA***A***, *n* = 1	2	1 belongs to *SMN1* 1 belongs to *SMN2*	A*T*GG*A*, *n* = 2	2	2	*SMN1* variant: c.*239G>A; 1 copy of *SMN1* exon 8 converted to *SMN2* exon 8
YZ43	2	1	2	3	2	1	*GC*AA*G*, *n* = 1 *GC*AA***A***, *n* = 1	2	1 belongs to *SMN1* 1 belongs to *SMN2*	A*T*GG*A*, *n* = 2	2	2	*SMN1* variant: c.*239G>A; 1 copy of *SMN1* exon 8 converted to *SMN2* exon 8
YZ44	2	1	1	1	2	1	G*C*AA*G*, *n* = 2	2	2	A*T*GG*A*, *n* = 1	1	1	MLPA could only detect 1 copy of *SMN1* exon 8 as the *SMN1* variant c.*237_*238del disturb the combination between *SMN1* exon 8 and relevant MLPA probe.

^a^PSVs, paralogous sequence variants; Exon 7 with C at locus c.840 (the second PSV, italic format) and exon 8 with G at locus c.*239 (the last PSV, italic format) belong to *SMN1*; Exon 7 with T at locus c.840 (the second PSV, italic format) and exon 8 with A at locus c.*239 (the last PSV, italic format) belong to *SMN2*

^b^Consistent results across all three testing methods

### Samples with inconsistent MLPA and qPCR results

For *SMN1* exon 7, the results determined by MLPA and qPCR were inconsistent in 16 samples (Table 2, results of MLPA and qPCR). CASMA was performed on these samples to quantify copy numbers and analyze the possible reasons for the inconsistency between MLPA and qPCR detection results in the first experiment (Table 2, Results of CASMA). We also recollected samples for MLPA and qPCR. It was interesting that the second quantification results of *SMN1* exon 7 by both methods were all consistent with those of CASMA, except for an *SMN1* variant c.-39A>G was additionally identified by CASMA in one sample (Fig. 1B). The quantitative inconsistencies of 10 samples were related to *SMN1* exon 7 copy number duplications (three or four copies), with MLPA results in five samples located in the gray area of the data, qPCR results in one sample located in the gray area of the data, and the other four qPCR results being false-negative results for copy number duplications. For the heterozygous deletion variant (one copy), MLPA detected no false-negative or false-positive results, whereas qPCR detected six false-positive results (Table 2).

**Table 2:** CASMA analysis of samples with inconsistent MLPA and qPCR results

Sample	MLPA				qPCR		CASMA				Comments
	*SMN1* Exon 7	*SMN1* Exon 8	*SMN2* Exon 7	*SMN2* Exon 8	*SMN1* Exon 7	*SMN1* Exon 8	*SMN1*	Specific PSVs	*SMN2*	Specific PSVs	
RT01	2 (2 ~ 3, NLT)	2 (2 ~ 3, NLT)	1	1	1	2	2	G*C*AA*G*, *n* = 2	1	A*T*GG*A*, *n* = 1	False-positive results of qPCR for detection of *SMN1* heterozygous deletion
RT02	2	2	2	2	1	2 (2 ~ 3, NLT)	2	G*C*AA*G*, *n* = 2	2	A*T*GG*A*, *n* = 2	
RT03	2	2	1	1	1	2	2	G*C*AA*G*, *n* = 2	1	A*T*GG*A*, *n* = 1	
RT04	2	2	1	1	1	2	2	G*C*AA*G*, *n* = 2	1	A*T*GG*A*, *n* = 1	
RT05	2	2	1	1	1 (1 ~ 2, NLT)	2	2	G*C*AA*G*, *n* = 2	1	A*T*GG*A*, *n* = 1	
RT06	2	2	2	2	1 (1 ~ 2, NLT)	2	2	G*C*AA*G*, *n* = 2	2	A*T*GG*A*, *n* = 2	
RT07	3	3	0	0	2 (2 ~ 3, NLT)	2	3	G*C*AA*G*, *n* = 3	0	A*T*GG*A*, *n* = 0	False-negative results of qPCR for detection of *SMN1* heterozygous duplication
RT08	3	3	1	1	2	3 (2 ~ 3, NUT)	3	G*C*AA*G*, *n* = 3	1	A*T*GG*A*, *n* = 1	
RT09	3	3	1	1	2	2	3	G*C*AA*G*, *n* = 3	1	A*T*GG*A*, *n* = 1	
RT10	3	3	1	1	2	3	3	G*C*AA*G*, *n* = 3	1	A*T*GG*A*, *n* = 1	
RT11	3	3	1	1	2	3	3	G*C*AA*G*, *n* = 3	1	A*T*GG*A*, *n* = 1	
RT12	2 (2 ~ 3, NLT)	2 (2 ~ 3, NLT)	1	1	3	3	3	G*C*AA*G*, *n* = 3	1	A*T*GG*A*, *n* = 1	False-negative results of MLPA for detection of *SMN1*duplications
RT13	2 (2 ~ 3, NLT)	2 (2 ~ 3, NLT)	1	1	3	3 (2 ~ 3, NUT)	3	G*C*AA*G*, *n* = 3	1	A*T*GG*A*, *n* = 1	
RT14	2 (2 ~ 3, NLT)	2 (2 ~ 3, NLT)	2	2	3 (2 ~ 3, NUT)	3	3	G*C*AA*G*, *n* = 3	2	A*T*GG*A*, *n* = 2	
RT15	2 (2 ~ 3, NLT)	2 (2 ~ 3, NLT)	2	2	3	2	3	G*C*AA*G*, *n* = 3	2	A*T*GG*A*, *n* = 2	
RT16	2 (2 ~ 3, NLT)	2 (2 ~ 3, NLT)	2	2	4	4	4	G*C*AA*G*, *n* = 4	2	A*T*GG*A*, *n* = 2	

NLT, the copy number is near the lower threshold; NUT, the copy number is near the upper threshold

### Overall statistics of the selected samples

In this study, 16 samples had inconsistent MLPA and qPCR results. After supplemental CASMA verification and re-sampling for detection by MLPA and qPCR, we collected and summarized the final MLPA test results in Table 3. In the selected population for this study, the frequencies of *SMN1* exon 7 in one, two, three, and four copies were 1.64%, 93.79%, 4.43%, and 0.14%, respectively. For *SMN2*, the copy number frequencies of exon 7 in zero, one, two, and three copies were 4.71%, 36.57%, 55.50%, and 3.21%, respectively. A total of 23 SMA carriers were detected, with a prevalence of 1/61 and a detection rate of 1.64%. Fourteen carriers were formed due to *SMN1* deletion (1.00%), among which four samples with *SMN1*: *SMN2* = 1:1 and 10 samples with *SMN1*: *SMN2* = 1:2. Nine carriers were formed due to the conversion of *SMN1* to *SMN2* (0.64%), with the genotype *SMN1*: *SMN2* = 1:3. No SMA carrier, with genotype *SMN1*: *SMN2* = 1:0 was detected. Homozygous deletion of *SMN2* was detected in 66 samples, accounting for 4.71% of the total.

**Table 3:** Copy number status of *SMN1*/*2* exons 7 and 8 detected in this study

MLPA SMN1		MLPA SMN2		Sample (*n*)	Ratio
Exon 7	Exon 8	Exon 7	Exon 8		
**1**	**1**	**1**	**1**	**4**	**0.29%**
**1**	**1**	**2**	**2**	**10**	**0.71%**
**1**	**1**	**3**	**3**	**9**	**0.64%**
2	1	1	1	1	0.07%
2	1	1	2	1	0.07%
2	1	2	2	2	0.14%
2	1	2	3	5	0.36%
2	2	0	0	64	4.57%
2	2	1	0	3	0.21%
2	2	1	1	455	32.50%
2	2	1	2	1	0.07%
2	2	2	1	4	0.29%
2	2	2	2	716	51.14%
2	2	3	2	1	0.07%
2	2	3	3	33	2.36%
2	3	1	0	8	0.57%
2	3	2	1	18	1.29%
2	3	2	2	1	0.07%
3	2	1	2	1	0.07%
3	3	0	0	2	0.14%
3	3	1	0	1	0.07%
3	3	1	1	35	2.50%
3	3	2	1	1	0.07%
3	3	2	2	19	1.36%
3	3	3	3	2	0.14%
3	4	1	0	1	0.07%
4	4	1	1	1	0.07%
4	4	2	2	1	0.07%
Total				1400	100.00%

### Analysis of the “2 + 0” genotype in a family

**Fig. 2:**
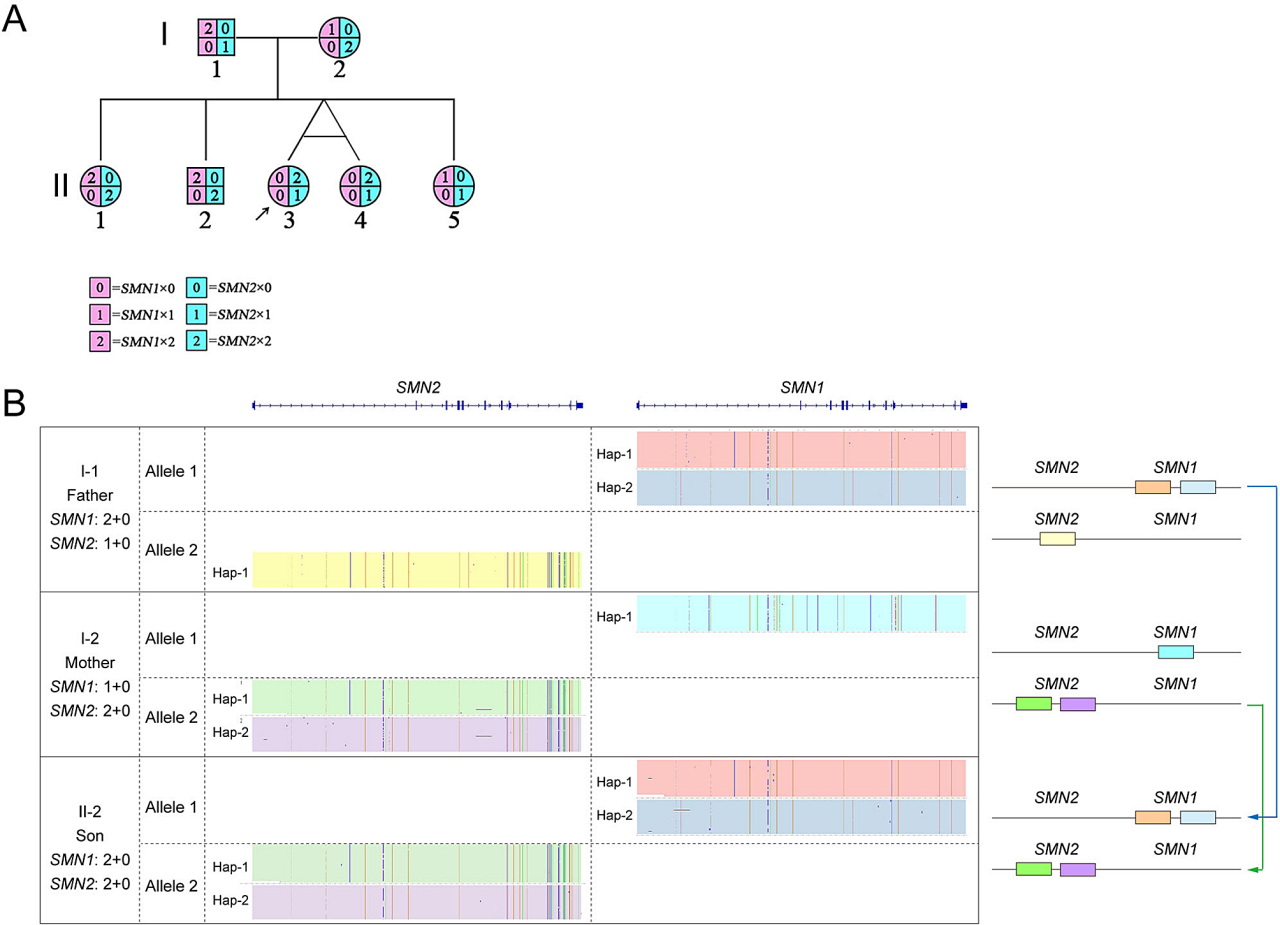
Diagram of the family pedigree and CASMA analysis. (**A**) *SMN1* and *SMN2* were highlighted in pink and cyan, respectively. Numbers within the pedigree were used to indicate the copy numbers of the gene. II-3 and II-4 were identical twins. (**B**) Distribution patterns of *SMN1* and *SMN2* on the two alleles detected by CASMA (I-1, I-2, and II-2). Arrows indicated the inheritance of alleles between generations

In the genetic diagnosis of suspected SMA patients, we collected a family. The proband had a homozygous deletion of the *SMN1* gene, the proband’s mother had a heterozygous deletion of the *SMN1* gene, and the copy number of the *SMN1* gene of the proband’s father was two. Therefore, the proband’s father was suspected to have the “2 + 0” genotype. The proband’s father’s genotype could not be determined by three-generation linkage analysis, as samples from the proband’s paternal grandparents were not available. There were two possibilities for the two copies of *SMN1* in the proband’s father: a carrier of the “2 + 0” genotype or a normal “1 + 1” genotype. Given that one of the four siblings of the proband had no *SMN1* copy, it is more likely that the proband’s father had a “2 + 0” genotype for *SMN1*. For the *SMN2* gene, through the similar genotype analysis described above, the *SMN2* genotype of the proband’s mother was likely to be “2 + 0”. Finally, we inferred that the parental genotypes of this family should be *SMN1* × 0 & *SMN2* × 1/*SMN1* × 2 & *SMN2* × 0 (the proband’s father, I-1) and *SMN1* × 1 & *SMN2* × 0/*SMN1* × 0 & *SMN2* × 2 (the proband’s mother I-2), respectively. The genetic pedigree is shown in Fig. 2A.

CASMA enables haplotype analysis by SNP data of the full-length sequence of *SMN1*/*2*. Using the haplotype analysis results of two generations, the “2 + 0” carriers can be finally identified/excluded. We selected three samples (I-1, I-2, and II-2) from this family to verify the ability of CASMA to analyze “2 + 0” carriers. According to the results of CASMA haplotype analysis (Fig. 2B), the two *SMN1* copies of II-2 were all inherited from his father. II-2 and his father I-1 were both *SMN1* “2 + 0” genotypes. The two *SMN2* copies of II-2 were all inherited from his mother. II-2 and his mother were both *SMN2* “2 + 0” genotypes. Compared with traditional PCR-based genetic testing methods, CASMA can determine the “2 + 0” genotype by genotyping only the parents and one child.

## Discussion

Quantitative analysis of the *SMN1* exon 7 copy number is the major strategy for carrier screening and prenatal diagnosis of SMA. Currently, the more widely used methods MLPA and qPCR focus on the targeted amplification of different nucleotide sequences in *SMN1* exon 7. Quantitative PCR is one of the most commonly used methods for carrier screening in the population due to its low cost and simple operation. However, false-positive results may occur due to non-specific amplification of DNA fragments. MLPA has high sensitivity and accuracy but is not suitable for large-scale carrier screening due to its high cost, complicated operation steps, and long testing period. Therefore, most clinical laboratories prefer to use qPCR for screening first and then use MLPA for validation. For samples with abnormal qPCR results, many laboratories use MLPA as the “gold standard” for verification. For samples that are negative by qPCR, most of them will be released as negative results. Regarding the reliability of these negative results, it is necessary to conduct a large-scale controlled experiment for verification. Tan et al. used qPCR, MLPA, droplet digital PCR (ddPCR), high-resolution melting (HRM) analysis, and PCR-based capillary electrophoresis (PCR/CE) to re-test *SMN1* copy numbers in 516 retrospective samples that had undergone SMA carrier screening (qPCR). The MLPA results were then used as a reference to compare the performance of these methods. Relative to other methods that showed 100% consistency with MLPA, the sensitivities of qPCR for detecting 1, 2, and >2 copies of *SMN1* exon 7 were 100%, 99.7%, and 96.3%, respectively [18]. However, these conclusions are all predicated on the premise that quantitative MLPA results are 100% reliable. There is only a one-nucleotide difference between *SMN1* and *SMN2* exon 7. Targeted tests for this region have some probability of false-negative and false-positive results. The results of MLPA also need to be scientifically evaluated.

In 2022, Li et al. developed CASMA assay to detect SMA variants based on a LRS platform, which detected SMA carriers carrying one copy of *SMN1* with a sensitivity and specificity of 100% and 99.2%, respectively. This method is expected to increase the detection rate of SMA carriers from 91 to 98% and reduce the residual risk ratio from 1/415 to 1/1868, showing important clinical utility and promise for application in carrier screening [16]. In this study, both qPCR and MLPA were used to test 1400 samples. Samples with inconsistent results were tested using CASMA and resampled for MLPA and qPCR. As shown in Table 2, MLPA may produce false-negative results when testing samples with an increased copy number of *SMN1* exon 7, although often near the threshold. In contrast, the detection of the *SMN1* copy number by qPCR produces both false-negative and false-positive results. Of the five cases in which the copy number of exon 7 should have been three, qPCR showed a copy number of two, with only one case in the gray area. The other six cases of false-positive results reported two copies of exon 7 as one copy. The amplification efficiency of MLPA probes is relatively easy to be consistent, as they are amplified with the same set of primers after ligation. Whereas, most qPCR assays use the reference gene comparison mode, where the amplification primers of the reference gene and the target gene are different, and their PCR amplification efficiency may lead to deviation. Comparatively speaking, MLPA data are more reliable, but with less than 100% accuracy. Therefore, its use as the “gold standard” for SMA testing is only relative. In addition, when analyzing the MLPA test results, we found 13 samples with unequal copy numbers of exons 7 and 8 in *SMN1* and/or *SMN2*, which could be confused. We used CASMA for testing. Analysis of five different nucleotides between *SMN1* and *SMN2* genes showed that these copy number differences were due to the conversion of exon 8 between *SMN1* and *SMN2* (Fig. 1C and E) or the variant in the MLPA probe-binding region (Fig. 1F). We also found that homologous recombination between the highly homologous *SMN1* and *SMN2* occurs not only in exon 8 but also in exon 7 or its adjacent region (YZ33, Fig. 1D), which could only be revealed by CASMA analysis.

CASMA was performed on 60 samples in this study. For samples with consistent MLPA and qPCR results, the CASMA results showed 100% consistency and could intuitively explain the inconsistency of exons 7 and 8 copy numbers of MLPA. For samples with inconsistent results between MLPA and qPCR, CASMA avoids the influence of sample quality, experimental operation, data analysis, and other factors on the results, and obtains more accurate results. Therefore, based on the MLPA/qPCR sequencing results corrected by CASMA, the carrier rates of *SMN1* and *SMN2* in the selected population and the copy number distribution were closer to the real situation. As a special type of carrier, the carrier rate of the “2 + 0” genotype in the general population is about 5%~8% [5]. Quantitative genetic testing techniques such as MLPA and qPCR can test the copy number of *SMN1* and *SMN2* genes, but they are unable to distinguish between the “2 + 0” genotype and the normal “1 + 1” genotype. Genotype analysis of three generations of family members or two generations of families members with multiple children is often required to determine whether a person is a “2 + 0” carrier [19]. The clinical feasibility of this approach is limited by the requirement of multiple family members and the complexity of STR analysis. CASMA determined the “2 + 0” carrier status of *SMN1* in the father (I-1) and *SMN2* in the mother (I-2) by analyzing the genotypes of three samples from both parents and one sibling, re-emphasizing its simplicity, rapidity, accuracy, and effectiveness in the screening of “2 + 0” carriers. In addition, CASMA detected an additional intragenic variant c.-39A>G in *SMN1* in one sample (RT02) with two *SMN1* copies, which was first reported in a patient with SMA (compound heterozygous with an *SMN1* deletion) and may reduce normal protein expression by affecting translation [20].

An ideal carrier screening method for SMA should be cost-effective, high throughput, easy to perform and automated, and in particular good performance should come first. Although compared to the cost of qPCR and MLPA, CASMA seems to be more expensive (approximately $25). However, with the gradual reduction in the cost of LRS and the increased throughput of the new PacBio LRS platform, the cost of carrier screening method for SMA will be further reduced in the future. Therefore, CASMA has good potential for clinical application in the first-line carrier screening of SMA.

## Conclusions

In summary, CASMA can not only quantify the copy number of the *SMN* gene but also accurately detect intragenic variants and easily determine the “2 + 0” genotype of subjects. It is a simple and accurate screening method for SMA, which shows greater clinical efficacy in the large-scale screening of SMA.

## Data Availability

The data that support the findings of this study are available from the corresponding author on reasonable request.

## Abbreviations

SMA: Spinal muscular atrophy
MLPA: Multiplex ligation-dependent probe amplification
qPCR: Quantitative polymerase chain reaction
SMN: Survival motor neuron
SMN1: Motor Neuron Survival Gene 1
LRS: Long-read sequencing
CASMA: Comprehensive analysis of SMA
TGS: Third-generation sequencing
CCS: Circular consensus sequencing
IGV: Integrated Genomics Viewer
STR: Short tandem repeat
PSVs: Paralogous sequence variants
ddPCR: Droplet digital PCR
HRM: High-resolution melting
PCR/CE: PCR-based capillary electrophoresis

## References

[1] Mercuri E, Sumner CJ, Muntoni F, Darras BT, Finkel RS. Spinal muscular atrophy. Nat Reviews Disease Primers. 2022;8(1):52. 35927425 10.1038/s41572-022-00380-8

[2] Chaytow H, Huang YT, Gillingwater TH, Faller KME. The role of survival motor neuron protein (SMN) in protein homeostasis. Cell Mol Life Sci. 2018;75(21):3877–94. 29872871 10.1007/s00018-018-2849-1 PMC6182345

[3] Lorson CL, Hahnen E, Androphy EJ, Wirth B. A single nucleotide in the SMN gene regulates splicing and is responsible for spinal muscular atrophy. Proc Natl Acad Sci USA. 1999;96(11):6307–11. 10339583 10.1073/pnas.96.11.6307 PMC26877

[4] Prior TW, Nagan N, Sugarman EA, Batish SD, Braastad C. Technical standards and guidelines for spinal muscular atrophy testing. Genet Medicine: Official J Am Coll Med Genet. 2011;13(7):686–94. 10.1097/GIM.0b013e318220d523 21673580

[5] Verhaart IEC, Robertson A, Wilson IJ, Aartsma-Rus A, Cameron S, Jones CC, et al. Prevalence, incidence and carrier frequency of 5q-linked spinal muscular atrophy - a literature review. Orphanet J Rare Dis. 2017;12(1):124. 28676062 10.1186/s13023-017-0671-8 PMC5496354

[6] Mercuri E, Bertini E, Iannaccone ST. Childhood spinal muscular atrophy: controversies and challenges. Lancet Neurol. 2012;11(5):443–52. 22516079 10.1016/S1474-4422(12)70061-3

[7] Consensus Expert Group On Carrier Screening For Monogenic D, Genetic Counseling Group Of Medical Genetics Branch Of Chinese, Medical A, Hu T, Guo J, Liu S, Lu Y, et al. [Expert consensus over genetic counseling for carrier screening of spinal muscular atrophy]. Zhonghua Yi Xue Yi Chuan Xue Za Zhi = Zhonghua Yixue Yichuanxue zazhi = Chinese. J Med Genet. 2024;41(6):661–8. 10.3760/cma.j.cn511734-20231115-00256 38818549

[8] Mercuri E, Pera MC, Scoto M, Finkel R, Muntoni F. Spinal muscular atrophy - insights and challenges in the treatment era. Nat Reviews Neurol. 2020;16(12):706–15. 10.1038/s41582-020-00413-4 33057172

[9] Smith M, Calabro V, Chong B, Gardiner N, Cowie S, du Sart D. Population screening and cascade testing for carriers of SMA. Eur J Hum Genetics: EJHG. 2007;15(7):759–66. 17392705 10.1038/sj.ejhg.5201821

[10] Mailman MD, Hemingway T, Darsey RL, Glasure CE, Huang Y, Chadwick RB, et al. Hybrids monosomal for human chromosome 5 reveal the presence of a spinal muscular atrophy (SMA) carrier with two SMN1 copies on one chromosome. Hum Genet. 2001;108(2):109–15. 11281448 10.1007/s004390000446

[11] Sugarman EA, Nagan N, Zhu H, Akmaev VR, Zhou Z, Rohlfs EM, et al. Pan-ethnic carrier screening and prenatal diagnosis for spinal muscular atrophy: clinical laboratory analysis of > 72,400 specimens. Eur J Hum Genetics: EJHG. 2012;20(1):27–32. 21811307 10.1038/ejhg.2011.134 PMC3234503

[12] Feldkötter M, Schwarzer V, Wirth R, Wienker TF, Wirth B. Quantitative analyses of SMN1 and SMN2 based on real-time lightCycler PCR: fast and highly reliable carrier testing and prediction of severity of spinal muscular atrophy. Am J Hum Genet. 2002;70(2):358–68. 11791208 10.1086/338627 PMC419987

[13] Arkblad EL, Darin N, Berg K, Kimber E, Brandberg G, Lindberg C, et al. Multiplex ligation-dependent probe amplification improves diagnostics in spinal muscular atrophy. Neuromuscul Disorders: NMD. 2006;16(12):830–8. 17049859 10.1016/j.nmd.2006.08.011

[14] Mercuri E, Finkel RS, Muntoni F, Wirth B, Montes J, Main M, et al. Diagnosis and management of spinal muscular atrophy: part 1: recommendations for diagnosis, rehabilitation, orthopedic and nutritional care. Neuromuscul Disorders: NMD. 2018;28(2):103–15. 29290580 10.1016/j.nmd.2017.11.005

[15] Lefebvre S, Bürglen L, Reboullet S, Clermont O, Burlet P, Viollet L, et al. Identification and characterization of a spinal muscular atrophy-determining gene. Cell. 1995;80(1):155–65. 7813012 10.1016/0092-8674(95)90460-3

[16] Li S, Han X, Xu Y, Chang C, Gao L, Li J, et al. Comprehensive analysis of spinal muscular atrophy: SMN1 Copy Number, Intragenic Mutation, and 2 + 0 carrier analysis by third-generation sequencing. J Mol Diagnostics: JMD. 2022;24(9):1009–20. 35659528 10.1016/j.jmoldx.2022.05.001

[17] Bai J, Qu Y, Huang W, Meng W, Zhan J, Wang H, et al. A high-fidelity long-read sequencing-based approach enables accurate and effective genetic diagnosis of spinal muscular atrophy. Clin Chim Acta. 2024;553:117743. 38158006 10.1016/j.cca.2023.117743

[18] Tan J, Zhang J, Sun R, Jiang Z, Wang Y, Ma D, et al. Evaluating the performance of four assays for carrier screening of spinal muscular atrophy. Clin Chim Acta. 2023;548:117496. 37479010 10.1016/j.cca.2023.117496

[19] Yanyan C, Miaomiao C, Fang S, Yujin Q, Jinli B, Hong W. Familial study of spinal muscular atrophy carriers with SMN1 (2 + 0) genotype. Yi Chuan = Hereditas. 2021;43(2):160–8. 33724218 10.16288/j.yczz.20-319

[20] Wang CC, Chang JG, Chen YL, Jong YJ, Wu SM. Multi-exon genotyping of SMN gene in spinal muscular atrophy by universal fluorescent PCR and capillary electrophoresis. Electrophoresis. 2010;31(14):2396–404. 20564270 10.1002/elps.201000124

